# Insights into Allosteric Mechanisms of the Lung-Enriched p53 Mutants V157F and R158L

**DOI:** 10.3390/ijms231710100

**Published:** 2022-09-03

**Authors:** Jiangtao Lei, Xuanyao Li, Mengqiang Cai, Tianjing Guo, Dongdong Lin, Xiaohua Deng, Yin Li

**Affiliations:** 1 Institute of Space Science and Technology, Nanchang University, Xuefu Avenue 999, Nanchang 330031, China; 2 Department of Physics, School of Physics and Materials Science, Nanchang University, Xuefu Avenue 999, Nanchang 330031, China; 3 Department of Physics and Qian Xuesen Collaborative Research Center of Astrochemistry and Space Life Sciences, Ningbo University, Ningbo 315211, China

**Keywords:** p53, V157F mutant, R158L mutant, allosteric mechanism, molecule dynamic simulation

## Abstract

Lung cancer is a leading fatal malignancy in humans. p53 mutants exhibit not only loss of tumor suppressor capability but also oncogenic gain-of-function, contributing to lung cancer initiation, progression and therapeutic resistance. Research shows that p53 mutants V157F and R158L occur with high frequency in lung squamous cell carcinomas. Revealing their conformational dynamics is critical for developing novel lung therapies. Here, we used all-atom molecular dynamics (MD) simulations to investigate the effect of V157F and R158L substitutions on the structural properties of the p53 core domain (p53C). Compared to wild-type (WT) p53C, both V157F and R158L mutants display slightly lesser β-sheet structure, larger radius of gyration, larger volume and larger exposed surface area, showing aggregation-prone structural characteristics. The aggregation-prone fragments (residues 249–267 and 268–282) of two mutants are more exposed to water solution than that of WT p53C. V157F and R158L mutation sites can affect the conformation switch of loop 1 through long-range associations. Simulations also reveal that the local structure and conformation around the V157F and R158L mutation sites are in a dynamic equilibrium between the misfolded and properly folded conformations. These results provide molecular mechanistic insights into allosteric mechanisms of the lung-enriched p53 mutants.

## 1. Introduction

Lung cancer is the most often diagnosed cancer all over the world and is the most frequent cause of cancer death due to the high incidence of treatment failure [1]. Smoking is a major cause of lung cancer. Chronic exposure of the lung epithelium to tobacco smoke confers increased oncogenic mutation of tumor suppressor p53 [2]. Normally, as the “guardian of the human genome”, p53 regulates many critical cellular activities, such as apoptosis, cell cycle control, and damaged DNA repair, showing its tumor-suppressive capabilities [3,4,5]. However, when impacted by mutations, p53 not only loses its protective functions but also gains oncogenic effects [6,7], greatly leading to lung cancer initiation, progression, and therapeutic resistance [8]. 

p53 protein is composed of an N-terminus containing transactivation domains (residues 1–92), a DNA-binding domain (residues 94–312, referred to as p53C or DBD) that binds to specific DNA sequences, and a C-terminus oligomerization and regulatory domains (residues 313–393) [9,10,11]. p53 is mutated in more than half of human cancer, with more than 95% of the mutations occurring in DNA-binding regions [12]. Generally, the mutations in p53 are categorized roughly into two groups based on their mode of action [13]. The first group consists of DNA-contact mutations, such as R248Q, R273H, and R282W, which affect the domains that are directly involved in specific DNA binding. The second group consists of structural (also referred to as destabilizing or aggregating) mutations, such as R175H, Y220C, and R249S, which cause either a full or partial distortion of the correct folding of the p53C [14]. The structurally destabilizing mutants have more propensity than DNA-contact mutants to form amyloid-like aggregates. Mutant p53 aggregates trigger not only the co-aggregation of wild-type p53 (dominant-negative effect) [15] but also the cross-reaction of its two homologues p63 and p73 (gain-of-function effect) [16,17], which makes the cancer cells more aggressive [18,19]. 

Of particular interest are V157F and R158L mutants in p53, which occur with increased frequency in lung cancer, surpassing that of many traditional hotspots [20]. The increased occurrence of V157F and R158L mutations is attributed to preferential DNA adduct formation at these codons by carcinogenic polycyclic aromatic hydrocarbons in cigarette smoke [2,21]. The two mutants exhibit defective transactivation ability with less than 20% of wild-type activity on p53 response elements [22]. Meanwhile, they can regulate a gain of function transcriptome in lung cancer and may confer de novo function [23]. The molecular simulations of the Y220C-DNA complex have shown that amino acid V157 is an important correlation signal site in the Y220-DNA allosteric pathway [24]. This phenomenon implies that the V157F mutation site may affect the structure of DNA-binding regions of V157F mutant through long-range associations, thus altering the biological function of p53 protein. In addition, the presence of p53 amyloid aggregates in lung carcinoma directly links p53 aggregation to the occurrence of lung cancer [25]. Whether the two mutants V157F and R158L have a higher aggregation tendency than WT p53? Hence, it is meaningful to explain these phenomena through revealing their structural features.

Full-length p53 is a typically multidomain protein flanked by disordered segments. p53C presents as a structurally ordered domain, while N- and C-terminal regions are intrinsically disordered and partially ordered domains, respectively [26]. Research shows that p53C displays similar thermodynamic stability and amyloid properties to that of full-length p53 [6,27]. Therefore, it is widely believed that p53C is a good model to recapitulate the property of full-length [28,29]. In this work, we aimed at unraveling the mechanism by which V157F and R158L substitutions alter the structural stability of p53C. By using all-atom molecular dynamics (MD) simulations, we compared structural differences between WT p53C and two mutants V157F and R158L. Both V157F and R158L mutants display slightly lesser β-sheet structure, larger radius of gyration, larger volume, larger exposed surface area, and more flexibility of Loop 1 than WT p53C. The aggregation-prone fragments (residues 249–267 and 268–282) of two mutants are more exposed to water solution than that of WT p53C. These phenomena suggest that the two mutants have a higher aggregation tendency than WT p53C. Simulations also reveal differential conformational sampling of loop 1 (residues 113–124) between WT p53C and the two mutants, probably implying different abilities for genome binding. Moreover, we found that the local structure and conformation around the V157F and R158L mutation sites are in a dynamic equilibrium between the misfolded and properly folded conformations. In V157F system, the aromatic ring of residue F157 exhibits ‘up’ and ‘down’ conformational states, which correspond to experimental structures of the V157F mutant and its rescued variants, respectively [30]. In R158L system, the β6–β7 turn also shows ‘open’ and ‘close’ states due to the variation of the local salt bridge network. These results provide molecular mechanistic insights into allosteric mechanisms of the lung-enriched p53 mutants V157F and R158L, thus providing a basis and idea for the design of related drugs.

## 2. Results and Discussion

The convergences of our simulations were assessed by checking the time evolution of β-sheet probability, RMSD, the total number of water molecules within 0.35 nm of p53C, and the total contact number of p53 protein in WT p53C, V157F and R158L mutant systems. As shown in Supplemental Figure S1, these parameters rapidly increase or decrease within the first 100 ns, and achieve reasonable dynamic equilibrium after 600 ns, suggesting that the structures of p53C in three systems are stable at the last 400 ns. Unless specified, all the MD simulation results presented below are based on the last 400 ns (time = 600–1000 ns) simulation data.

### 2.1. Both V157F and R158L Mutants Exhibit Structural Features of Aggregation-Prone States

**Figure 1:**
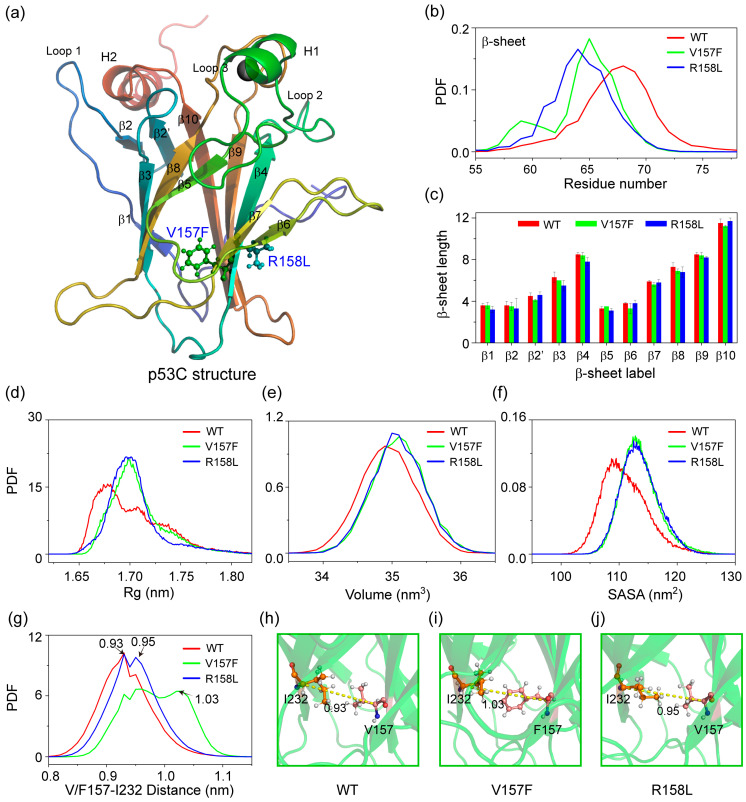
Structural analyses of p53C in WT, V157F and R158L systems. (**a**) The structure of p53C. The location of V157F and R158L mutations are marked. (**b**) The distribution of residue number for β-sheet structure over the MD runs for three systems. (**c**) The average β-sheet length (residue number) for eleven β-strands. The probability density function (PDF) of (**d**) the radius of gyration (Rg), (**e**) the volume, (**f**) the solvent-accessible surface area (SASA) of whole p53C, and (**g**) the distance between Cα atoms of V/F157 and I232. The snapshots of (**h**) WT, (**i**) V157F and (**j**) R158L systems at 1000 ns show the distance between Cα atoms of V/F157 and I232.

As seen in Figure 1a, p53C adopts an immunoglobulin-like β-sandwich fold with eleven β-strands (β1–β2, β2′–β10) and an extended DNA-binding surface, which is formed by a loop–sheet–helix motif (including loop 1: residues 113–124) and two large loops (loop 2: residues 163–194 and loop 3: residues 237–250) that are held together by zinc coordination [31]. The two lung-enriched V157F and R158L mutations locate on the β4 strand, which sits at the center of the β-sandwich fold. We first explored the effect of two mutations on the structure of p53C by counting the distribution of the residue number for β-sheet structure (Figure 1b). Compared to WT p53C, both two mutations promote a reduction in the formation of β-sheet structure. The β-sheet length of each strand is shown in Figure 1c. Most β-strands in V157F and R158L mutant systems tend to become slightly shorter than that of WT p53C. Figure 1d–f show that the average radius of gyration (Rg), volume and solvent-accessible surface area (SASA) of p53C in two mutant systems are larger than those in WT p53C. We further calculated the distance between Cα atoms of V/F157 and I232 shown in Figure 1g. The two mutations increase the local interlayer distance of the β-sandwich structure. These phenomena indicate that the two mutations cause the protein to swell and exhibit structural features of aggregation-prone molten-globule states [32,33]. We further assessed the internal interactions of p53C in three systems by calculating the number distribution of total contact, hydrogen bonds and salt bridges (Supplemental Figure S2a–c). These interactions are reduced due to the introduction of mutations. Meanwhile, the number of water molecules around p53C increases (Supplemental Figure S2d). These results show that V157F and R158L mutations reduce the stabilities of the two mutants and may facilitate their unfolding and aggregation by affecting the internal interactions.

**Figure 2:**
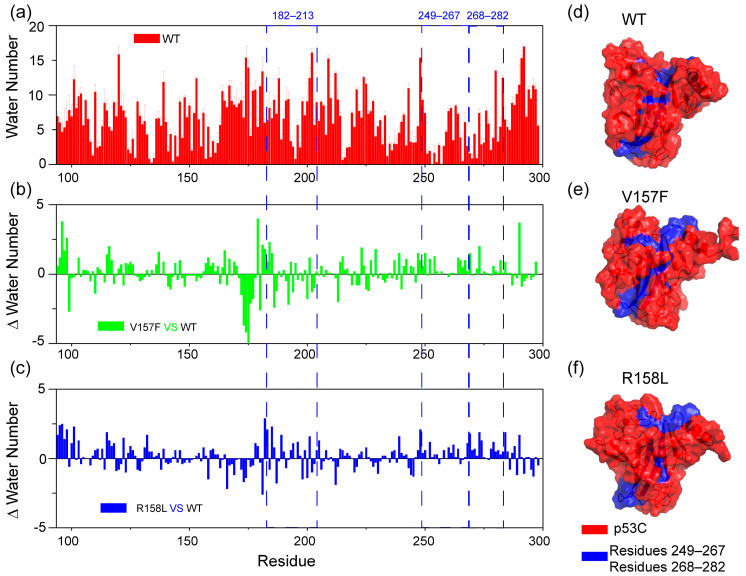
The extent of solvation of p53 C in WT, V157F and R158L systems. (**a**) The number of water molecules within 0.35 nm of WT p53C. The three aggregation core fragments (residues 182–213, 249–267 and 268–282) are pointed out by blue dotted line. (**b**,**c**) The difference value of water number between V157F/R158L mutant and WT p53C. (**d**–**f**) Snapshots of exposure for residues 249–267 and 268–282 in WT, V157F and R158L systems. Residues 249–267 and 268–282 are colored blue.

The exposure of some hydrophobic core regions in destabilized mutant p53 can trigger p53C to aggregate via forming an intermolecular β-sheet-like structure [13]. The experiments conducted by Wang et al. indicated that denatured p53 mutants may contain several aggregation-prone sequences and the first three main segments contributing to the aggregation process of p53 are residues 182–213, 249–267 and 268–282 [27]. To monitor the effect of the two mutations on the extent of solvation of p53C, we calculated the number of water molecules within 0.35 nm for each residue. As seen in Figure 2a–c, residues 249–267 and 268–282 in V157F and R158L mutant systems are more exposed than that in WT p53C, showing a higher potential to trigger aggregation. Snapshots of exposure for residues 249–267 and 268–282 are shown in Figure 2d–f. These results indicate that increasing the exposed regions (residues 249–267 and 268–282) may become a trigger for aggregation.

### 2.2. V157F and R158L Mutation Sites Affect the Conformation Switch of Loop 1 through Long-Range Associations

**Figure 3:**
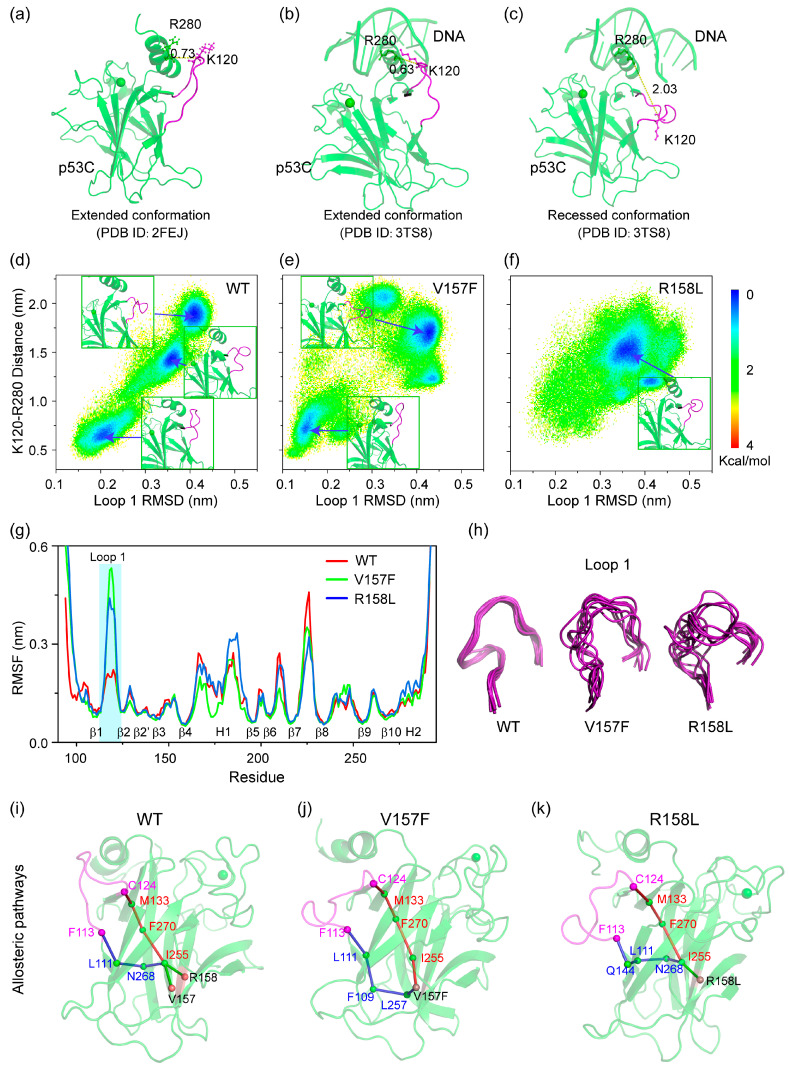
Effect of V157F and R158L mutations on the conformation and flexibility of loop 1. (**a**) The structure of WT p53C without DNA (PDB ID: 2FEJ). Loop 1 is colored magenta. The extended (**b**) and recessed (**c**) conformation of loop1 with DNA (PDB ID: 3TS8). Free energy surfaces (in kcal mol^−1^) of WT p53C (**d**), V157F (**e**) and R158L (**f**), respectively, as functions of loop 1 RMSD and the distance between Cα atoms of K120 and R280. (**g**) Average Cα-RMSF of p53C. (**h**) Snapshots show the different flexibilities of Loop 1. Nine representative snapshots at the simulation time = 600, 650, 700, 750, 800, 850, 950 and 1000 ns were superposed with each other. (**i**–**k**) Snapshots of optimal and suboptimal allosteric paths from residue V157 or R158 to loop 1, showing sphere and stick representations.

Crystal structures of p53-DNA complexes suggest that the sequence-specific DNA binding process is associated with a conformational switch in loop 1 of p53C [34]. Loop 1 adopts an extended conformation in the absence of DNA (Figure 3a). When bound to DNA as a tetramer, four p53 subunits show two distinct loop 1 conformations: an extended conformation for the inner subunits (Figure 3b) and a recessed conformation for the outer subunits (Figure 3c) [35,36]. We investigated the influence of two mutations on the conformational variation of loop 1 by constructing 2D free energy surface in Figure 3d–f using -RT ln H (loop 1 RMSD and K120-R280 distance) as described in analysis methods. The locations of representative structures are labeled on the PMF plot. For WT p53C, loop 1 presents three conformations (extended, recessed and recessed) located at (number of RMSD, distance) values of (0.2 nm, 0.7 nm), (0.37 nm, 1.4 nm) and (0.42 nm, 1.9 nm), respectively. The loop 1 conformations with values of (0.2 nm, 0.7 nm) and (0.42 nm, 1.9 nm) are very similar to extended and recessed conformations in the p53-DNA complex. The recessed conformation with values of (0.37 nm, 1.4 nm) may be an intermediate state between the extended and recessed conformations. Similar intermediate states of loop 1 have been observed experimentally in p53CR2–CONS26 structure [36]. For V157F mutant system (Figure 3e), though the extended and recessed conformations are observed, the intermediate state is lacking and loop 1 is more likely to form recessed conformation with lower energy. For the R158L mutant system, only the corresponding intermediate state appears. These phenomena imply that V157F and R158L mutations can affect the loop 1 conformation switch.

We further calculated the average Cα-RMSFs of p53C in three systems to investigate the influence of two mutations on conformational flexibility (Figure 3g). The eleven β-sheet regions show low flexibility, while loop regions show high flexibility in the WT p53C system. The V157F and R158L mutations mainly induce an increase in the flexibility of the loop 1 region. The enhanced flexibilities of loop 1 are intuitively observed in the superimposition of snapshots from different simulation times (Figure 3h). These results suggest that Loop 1 of V157F and R158L mutants become more unstable than WT p53C.

To unravel the mechanism of the long-range correlation between two mutation sites and loop 1, we determined the allosteric pathways from the mutation sites to loop 1. For each system, we select the mutation sites (residue 157 and 158) as the starting nodes and the residues of loop 1 as the ending nodes. Optimal and suboptimal paths between the starting and ending nodes are shown in Figure 3i–k. As seen in Figure 3i, the optimal and suboptimal paths from residues V157 to loop 1 are the same as the paths from residues R158 to loop 1 in WT system due to two residues are close to each other in sequence. Starting from V157 or R158, the signal propagates first to residue I255 and then split into two paths, which propagate separately to the N (pathway: I255-N268-L111-F113) and C (pathway: I255-F270-M133-C124) terminal of loop 1. In V157F and R158L mutant systems, the signal pathways to the C terminal are the same as that of WT p53C, while the other pathways to the N terminal change significantly. The variation of allosteric pathways suggests that V157F and R158L mutation mainly affect the loop 1 conformation via the long-range correlation between mutation sites and the N terminal of loop 1.

### 2.3. The Aromatic Ring of Residue F157 Emerges ‘Up’ and ‘Down’ Conformational States in V157F System

**Figure 4:**
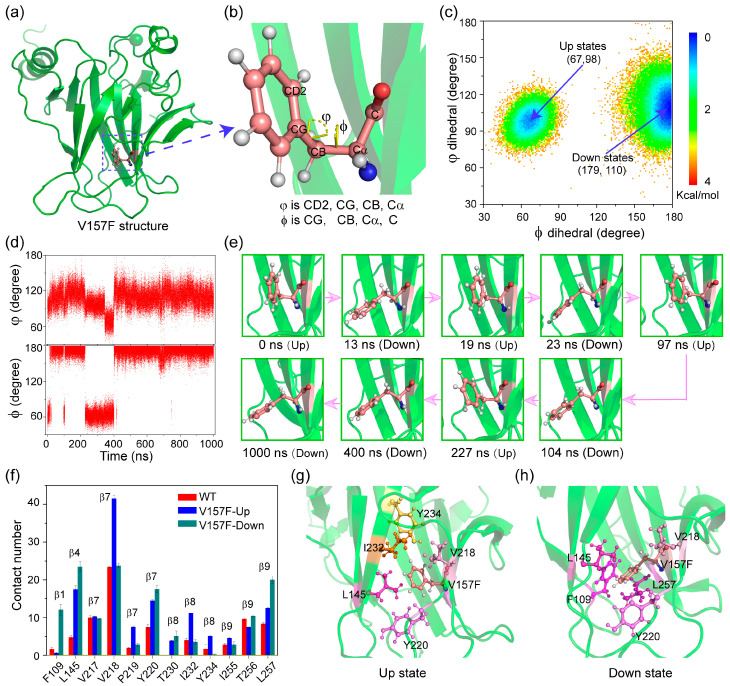
The orientation of aromatic ring of residue F157. (**a**) The structure of V157F mutant. (**b**) The local magnified snapshot of the aromatic ring of residue F157. Its orientation is described by two dihedral angles (φ, ϕ). (**c**) Potential mean force (PMF) as functions of dihedral angles φ and ϕ, emerging “up” and “down” conformational states in V157F system. (**d**) The function of two dihedral angles (φ, ϕ) with simulation time. (**e**) Snapshots of conformational states of the aromatic ring along simulation time. (**f**) The contact number between V157F and nearby amino acids. Snapshots of the interaction network in ‘up’ (**g**) and ‘down’ (**h**) conformational states.

Local structural changes at mutation sites are considered as an important factor destabilizing the overall structure of mutants. Here, we used two dihedral angles (φ, ϕ) to describe the orientations of the phenyl group of F157 (Figure 4a,b), where φ is the CD2-CG-CB-Cα dihedral angle and ϕ is the CG-CB-Cα-C dihedral angle. Potential mean force (PMF) as functions of dihedral angles φ and ϕ is shown in Figure 4c. Interestingly, there are two lowest energy potential wells, indicating two different states. The states with values (φ, ϕ) of (67, 98) and (179, 110) are referred to as ‘up’ and ‘down’ states, respectively. In the ‘up’ state, the phenyl group is oriented towards the interior of the hydrophobic core, consistent with the experimental structure of oncogenic mutant V157F (PDB ID: 4KVP) [30]. In the ‘down’ state, F157 points toward the edge of the β-sandwich, consistent with the rescued cancer mutant V157F/N235K/N239Y (PDB ID: 4LOF) [30]. The presence of two states possibly leads to an increase in the local interlayer distance of the β-sandwich structure (Figure 1g). As seen in Figure 4d,e, we further tracked the evolution of the phenyl group’s orientation over simulation time. The orientation can change rapidly between ‘up’ state and ‘down’ state via automatically crossing the energy barrier. Combining with the experimental phenomenon [30], we suggest that V157F mutant is in a dynamic equilibrium between the misfolded and properly folded conformations, instead of resting on the misfolded conformation.

To reveal the allosteric mechanism of the phenyl group of F157, the contact number between V157F and nearby amino acids was calculated in Figure 4f. Compared to WT p53C, the V157F mutation significantly enhances its interaction with other hydrophobic amino acids, probably due to the increased volume and hydrophobicity of the side chain. These interactions are directly involved in different β-strands. The large phenylalanine protrudes across the β-sandwich through strands β7 and β8 towards the surrounding hydration shell. The amino acids L145, V218 and Y220 have the highest contact number with F157 in the ‘up’ state, while amino acids F109, L145, V218, Y220 and L257 have the highest interactions with F157 in the ‘down’ state. Increased interactions between residues P219, I232, Y234 and residue F157 make the phenyl group orient towards the ‘up’. Increased interactions between residues F109, L145, Y220 and L257 and mutant residue F157 play a key role in the ‘down’ state. Snapshots of the interaction network in the ‘up’ (g) and ‘down’ (h) conformational states are shown in Figure 4g and h. The varied interaction network is likely to account for the orientation change in the aromatic ring.

### 2.4. The β6- β7 Turn Shows ‘Open’ and ‘Close’ States in R158L System Due to the Variation of the Local Salt-Bridge Network

**Figure 5:**
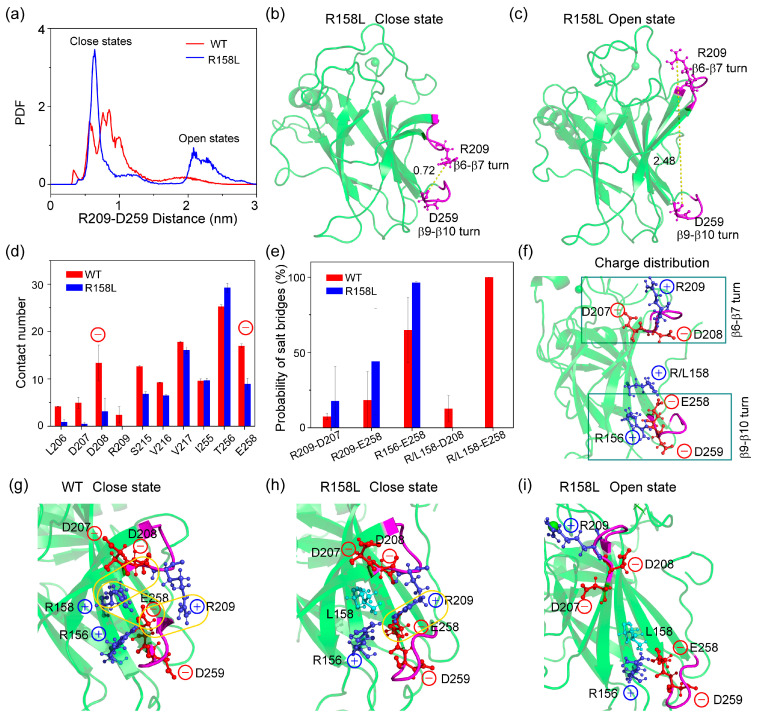
Differential conformational sampling of β6–β7 turn in R158L mutant system. (**a**) The distribution of the distance between CZ atom of R209 (β6–β7 turn) and the backbone carbonyl oxygen atom of D259 (β9–β10 turn). The snapshots of ‘close’ (**b**) and ‘open’ (**c**) states from two MDs at 1000 ns. (**d**) The contact number between R158L and nearby amino acids. (**e**) The salt-bridge probabilities (**f**) Initial charge distribution for β6–β7 turn and β9–β10 turn at WT and R158L mutant system. Charge positively residue R158 plays a key role in the salt-bridge network. (**g**) Salt-bridge network of WT p53C at 1000 ns. Salt-bridge networks of R158L mutant for ‘close’ (**h**) and ‘open’ (**i**) states at 1000 ns.

It is striking that simulations of WT and R158L mutant show distinct conformations of the β6–β7 turn, which is quantified by measuring the distance between the CZ atom of R209 on the β6–β7 turn (residues 208–213) and the backbone carbonyl oxygen of D259 on the β9–β10 turn (residues 259–263) [28]. In Figure 5a, these distance distributions across the simulations of the WT and R158L mutant peak at ∼0.5–1.0 nm (‘closed’ states) and ∼2.0–3.0 nm (‘open’ states) respectively. The ‘open’ and ‘close’ states of β6–β7 turn in R158L systems are shown in Figure 5b,c. The ‘open’ state of β6–β7 turn is a general feature of other destabilizing mutants including V143A, E258V, R110L, R175H and R248Q. [28] However, differently to those mutants, R158L still maintains the wild-type buried states (‘close’ states) with a higher probability than mutant solvent-exposed states (‘open’ states) (Figure 5a). These results suggest that the conformations of β6–β7 turn in R158L mutant are in a dynamic equilibrium between the misfolded and properly folded conformations, similar to the orientation of F157 in V157F mutant.

To probe the formation mechanisms of two distinct β6–β7 turn states, we compared the contact numbers between R158L and nearby amino acids in WT and R158L systems. In WT system (Figure 5d), residue R158 can interact with residues 206–209, 215–217, 255–256 and 258. When positively charged arginine is mutated to hydrophobic leucine, these involved residues (except for residue T256) reduced their interactions with L157, especially negatively charged residues D208 and E258. It is noted that D208 locates in β6–β7 turn region (residues 208–213) and E258 directly links the β9–β10 turn (residues 259–263). Decreased interactions between D208/E258 and R/L158 are against the formation of ‘closed’ states. In R158L system (Figure 5e), the probability of R209-D207, R209-E258 and R156-E258 salt bridges increases, while the salt bridges between D208/E258 and mutant site 158 disappear. The charge distribution of β6–β7 and β9–β10 turns are shown in Figure 5f. The β6–β7 and β9–β10 turn regions are electronegative. Thus, positively charged R158 in the middle plays a key role in a more buried state (‘closed’). As seen in Figure 5g, in the ‘close’ state of WT p53C, R158-D208, R158-E258 and R209-E258 can form salt bridges. In the ‘close’ state of R158L mutant, only R209-E258 salt bridge is maintained. In the ‘open’ state of R158L mutant, R158-D208, R158-E258 and R209-E258 salt bridges are lost. These phenomena indicate that the variation of the local salt-bridge network is a major reason for ‘open’ and ‘close’ states of β6–β7 turn in R158L system.

## 3. Materials and Methods

### 3.1. WT p53C, V157F and R158L Mutants

We investigated the structural properties of WT p53C, V157F and R158L mutant monomer. The initial coordinates of WT p53C and V157F were obtained from the PDB ID: 2FEJ [10] and ID: 4KVP [30], respectively. The fragment (residues 94–297) was chosen as a model system to maintain consistency with the experimental structure (PDB ID: 2FEJ), which contains the completely ordered region of the DNA-binding domain. The structure of V157F mutant superimposes well with WT p53C (Supplemental Figure S3) and the backbone RMSD between them is approximately 0.17 nm, showing a small structural deviation. Considering the absence of the experimental structure of R158L mutant, its starting state was generated by mutating the residue R158 at corresponding sites of WT p53C. To mimic the uncharged state of the two terminus residues in the full-length protein, the N-terminus and C-terminus of p53C were capped by acetyl (ACE) and amine (NH2), respectively. Histidine with protonation on ND1 were residues 178, 214 and 233, while those with protonation on NE2 were residues 115, 168, 179 and 193 [28]. We adopted the bonded model of Zinc [37] and covalently bonded it to residues Cys176(SG), Cys238(SG), Cys242(SG) and His 179(ND1).

### 3.2. Simulation Details

Three individual 1 *µ*s long MD simulations were performed for each protein system (WT p53C, V157F and R158L mutants) using the AMBER99SBILDN force field [38], which has been widely used in the research of p53 protein [39,40,41]. p53C protein is placed in a box filled with TIP3P water, with a minimum distance of 1.2 nm between the protein and the box edges. To keep the charge neutrality, neutralizing ions Na^+^ and Cl^−^ were added to each system. All simulations were performed using the GROMACS-9.1.3 software package in the NPT ensemble. The electrostatic interactions were calculated using the particle mesh Ewald (PME) method with a real space cut-off of 1.0 nm [42]. The pressure was kept at 1 bar by using the Parrinello–Rahman method [43] and the temperature was maintained at 310 K by using a velocity-rescaling coupling method [44]. The van der Waals interactions were treated using a cut-off of 1.0 nm. Constraints were applied to all-bond lengths using the Settle algorithm [45] for water molecules and the LINCS method [46]. The integration time step is 2 fs.

Trajectory analysis was carried out using the facilities implemented in the GROMACS-9.1.3 software package and our in-house codes. The DSSP program was used to calculate the secondary structure of p53C [47]. Gromacs tools were used to analyze the backbone root-mean-square-derivation (RMSD), the Cα root-mean-square-fluctuation (RMSF), the solvent-accessible surface area (SASA) and the number of hydrogen bonds. Our in-house codes were used to calculate the residue-residue contact number, the probability of salt bridge, the number of water molecules, and the volume of protein. Here, a contact was considered if the distance between two carbon atoms of nonsequential residues lies within 0.54 nm or the distance between any other two atoms of nonsequential residues lies within 0.46 nm [48,49,50]. A salt bridge was formed if the distance between the charge center of the charged side chain of four residues (including ARG+, LYS+, GLU− and ASP−) is within 0.4 nm [51]. The water number was counted if the distance between water molecules and protein is within 0.35 nm [39]. The volume of protein was calculated by the Monte Carlo algorithm [52]. The free energy surface of each system was constructed using −RT ln H(x, y) [53], where H(x, y) is the histogram of two selected reaction coordinates.

The allosteric signal transmission from the mutation sites to the allosteric area is analyzed by calculating the correlation pathways between mutation sites to the allosteric area. Atoms belonging to one amino acid are represented by a single node centered at the Cα atoms. An edge is assigned to a pair of nodes if the corresponding residue has a contact probability >70%. The weight of each edge is defined as W_ij_ = −log|C_ij_|, where C_ij_ stands for the dynamical cross correlation of two nodes (i and j). The length of a path D_ij_ between distant nodes i and j is defined as the sum of the edge weights between the consecutive nodes k, l along the path: D_ij_ = ∑_k,l_W_kl_. The optimal path between node i and j with the shortest network distance is found by the Floyd–Warshall algorithm [54,55]. 

## 4. Conclusions

In summary, we investigated the conformational and dynamic properties of WT p53C and the lung-enriched mutants V157F and R158L by performing multiple all-atom explicit solvent MD simulations. V157F and R158L mutants display slightly lesser β-sheet structure, larger radius of gyration, larger volume, larger exposed surface area and more flexibility of Loop 1 than WT p53C. The two aggregation-prone fragments (residues 249–267 and 268–282) of the two mutants are more exposed to water solution than that of WT p53C. These phenomena suggest that V157F and R158L mutations disrupt the stabilities of p53C and reduce shielding from the solvent, probably facilitating their unfolding and aggregation. The conformational switch in loop 1 of p53C allows DNA binding off-rates to be regulated independently of affinities [36]. The differential conformational samplings of Loop 1 in V157F and R158L systems probably imply different abilities for genome binding. Moreover, the local structure and conformation around the V157F and R158L mutation sites are in a dynamic equilibrium between the misfolded and properly folded conformations. In V157F system, the aromatic ring of residue F157 exhibits ‘up’ and ‘down’ conformational states, which correspond to experimental structures of the V157F mutant and its rescued variants, respectively [26]. In R158L system, the β6–β7 turn also shows ‘open’ and ‘close’ states due to the variation of the local salt bridge network. Considering these phenomena, we suggest that small-molecule or peptide drugs might stabilize the structure of V157F and R158L mutants by binding preferentially to the mutants when adopting a wild-type conformation and then gradually shifts the population equilibrium towards the wild-type states. These results provide molecular mechanistic insights into allosteric mechanisms of the lung-enriched p53 mutants V157F and R158L, thus providing a basis and idea for the design of related drugs.

## Data Availability

Not applicable.

## Supplementary Materials

The following supporting information can be downloaded at: https://www.mdpi.com/article/10.3390/ijms231710100/s1.
