# Supplementary material for: Insights into Allosteric Mechanisms of the Lung-Enriched p53 Mutants V157F and R158L
Source: Int J Mol Sci. 2022 Sep 3;23(17):10100. doi: 10.3390/ijms231710100 (PMC9456101; doi:10.3390/ijms231710100)
Supplement: Supplementary file 1 [file ijms-23-10100-s001.zip › ijms-1869907-supplementary.pdf]

This material contains three supplemental figures.

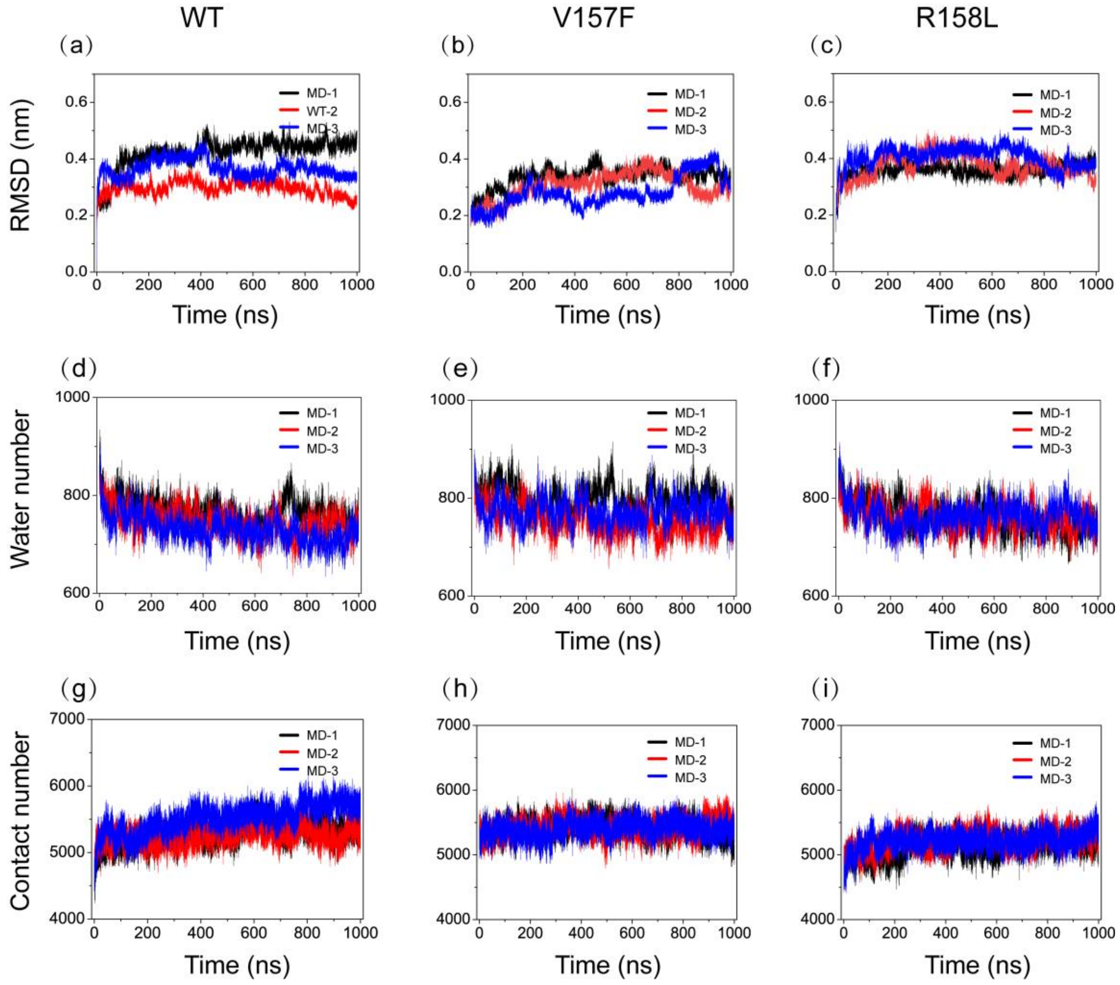

Figure S1. Convergence analysis of WT, V157F and R158L systems. (a-c) Time evolution of backbone root-mean-square-deviation (RMSD), (d-f) the total number of water molecules within 0.35 nm of p53C, (g-i) the contact number of whole p53C.

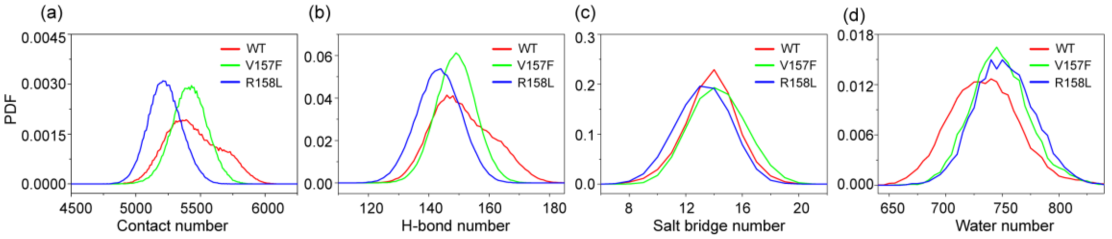

Figure S2. The probability density function (PDF) of (a) contact number, (b) the total number of hydrogen bonds (H-bond) (c) total salt-bridge number of p53C, (d) the total number of water molecules within 0.35 nm of p53C.

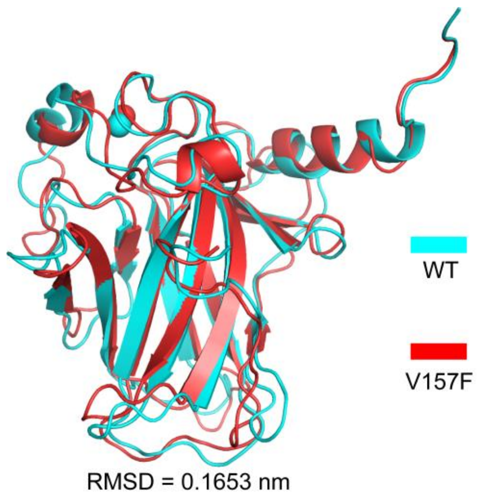

Figure S3. The superposed core domain structures of the initial conformations of WT and V157F.
